# Retinal Astrocytes Pretreated with NOD2 and TLR2 Ligands Activate Uveitogenic T Cells

**DOI:** 10.1371/journal.pone.0040510

**Published:** 2012-07-10

**Authors:** Guomin Jiang, Deming Sun, Henry J. Kaplan, Hui Shao

**Affiliations:** 1 Department of Ophthalmology and Visual Sciences, Kentucky Lions Eye Center, University of Louisville, Louisville, Kentucky, United States of America; 2 Doheny Eye Institute, Keck School of Medicine of the University of Southern California, Los Angeles, California, United States of America; Oregon Health & Science University, United States of America

## Abstract

On entering the tissues, infiltrating autoreactive T cells must be reactivated locally to gain pathogenic activity. We have previously reported that, when activated by Toll-like receptor 3 (TLR3) and TLR4 ligands, retinal astrocytes (RACs) are able to function as antigen-presenting cells to re-activate uveitogenic T cells and allow responder T cells to induce uveitis in mice. In the present study, we found that, although the triggering of TLR2 or nucleotide-binding oligomerization domain receptor 2 (NOD2) alone did not activate RACs, their combined triggering induced RACs with the phenotypes required to efficiently re-activate interphotoreceptor retinoid-binding protein (IRBP)-specific T cells. The synergistic effect of TLR2 and NOD2 ligands on RAC activation might be explained by the observations that bacterial lipoprotein (BLP, a TLR2 ligand) was able to upregulate NOD2 expression and the combination of BLP and muramyldipeptide (MDP, a NOD2 ligand) enhanced the expression of RICK (Rip2), the signaling molecule of NOD2. Moreover, the synergistic effect of MDP and BLP on RACs was lost when the RACs were derived from NOD2 knockout mice or were pre-treated with Rip2 antagonist. Thus, our data suggest that exogenous or endogenous molecules acting on both TLR2 and NOD2 on RACs might have an enhancing effect on susceptibility to autoimmune uveitis.

## Introduction

Although the exact etiology of non-infectious autoimmune uveitis remains unclear, bacterial and viral infections are potential cofactors implicated in the initiation and persistence of autoimmune diseases [1]. Accordingly, autoimmune uveitis, like ankylosing spondylitis, sarcoidosis, Behçet's disease, and inflammatory bowel disease, is frequently associated with previous bacterial infections [2].

A model of autoimmune uveitis, experimental autoimmune uveitis (EAU), can be induced in mice by immunization with interphotoreceptor retinoid-binding protein (IRBP) peptides in Freund's adjuvant containing heat-killed M. tuberculosis [3] and has been widely used to study the mechanisms underlying autoimmune uveitis. Studies on uveitis in man and animals have demonstrated that genetically predisposed individuals show a higher incidence of uveitis following exposure to an environmental trigger that activates uvea or retina-specific T cells [4], [5]. The peripheral activation of the autoreactive T cells allows them to cross the blood-retina barrier more easily, but, once in the tissues, these T cells must be reactivated locally to gain pathogenic activity [1], [6], [7], a process that relies on antigen presentation by antigen-presenting cells (APCs). However, the source of the APCs that re-activate infiltrating auto-reactive T cells in the eye is unclear. Infiltrating and resident macrophages and dendritic cells (DCs) in the eye might play a major role in this process [8]–[11]. Ocular parenchymal cells, such as retinal pigmental epithelium (RPE) cells and glia (microglia and astrocytes), also have the potential to act as APCs, especially when they are activated [12]–[15]. Activated parenchymal cells express MHC molecules, costimulatory molecules and cytokines, which, together, provide the necessary signals for the re-activation of infiltrating antigen-specific T cells.

As we recently reported [15], Toll-like receptor (TLR) ligands, commonly provided by pathogens, can activate retinal astrocytes (RACs), allowing them to present antigen for T cell re-activation. However, RACs show different responses to the triggering of different TLRs, resulting in qualitative and quantitative differences in the surface expression of costimulatory molecules and production of cytokines, which then induce different T cell responses [15]. In particular, a TLR3 ligand, polyinosine-polycytidylic acid (poly IC), and a TLR4 ligand, lipopolysaccharide (LPS) were found to be very effective in activating RACs, leading to the production of cytokines of both the Th1- and Th17-types that induce uveitis in naïve mice, whereas a TLR2 ligand, BLP, also called Pam3CSK4, was much less active.

Like TLRs, nucleotide-binding oligomerization domain (NOD)-like receptors belong to the family of pathogen recognition receptors (PRRs). NOD1 recognizes the dipeptide γ-D-glutamyl-*m*-diaminopimelic acid (iE-DAP) [16], [17], while NOD2 recognizes muramyl dipeptide (MDP) [18] and both iE-DAP and MDP are major components of peptidoglycan (PGN) present in Gram-negative and Gram-positive bacteria. Similar to TLRs, NODs are mainly expressed by DCs/macrophages and epithelial cells that are in direct contact with microbial organisms. Ligand binding causes NOD receptor oligomerization, exposure of the effector binding domain and the recruitment of receptor-interacting serine/threonine kinase (RICK), also known as RIP2, leading to activation of either the NF-kB pathway or the MAP kinase pathway and culminating in an inflammatory response mediated by IL-6, TNF-α, IL-12, IL-8, CXCL1, CXCL2, and CCL5 [19]–[21]. Recently, NOD1 and NOD2 were reported to be expressed in the eye and to be responsible for ocular inflammation in mice [22]–[24]. NOD2 mutation is implicated in Blau, a rare autosomal dominant disorder characterized by early-onset granulomatous arthritis, uveitis and skin rash with camptodactyly [25].

**Figure 1:**
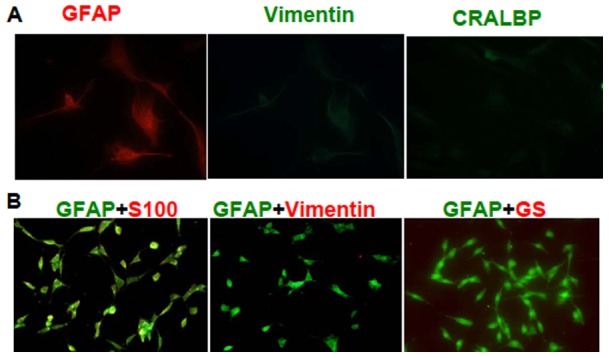
Characterization of RAC cultures. RACs from B6 mice were prepared as described in Materials and Methods. Before use, RAC were single-color stained with mAbs against GFAP (red), vimentin (green) and CRALBP (green) followed by immunofluorescence microscopy evaluation (A, 40 magnifications). The RACs were also two-color stained with mAbs against GFAP (green) and S-100 (red), GFAP and vimentin (red) or GFAP and GS (red) (B, 20 magnifications).

PGN is a major component of bacterial cell walls and can be recognized by both extracellular PRRs, such as TLR2, and intracellular PRRs, such as NOD1/NOD2 [26]; [27]. However, an interaction between NOD proteins and TLR2 remains controversial. Since NOD proteins and TLRs can be activated by the same microbial organisms, leading to the activation of the same signaling pathway that results in the production of pro-inflammatory cytokines and antimicrobial peptides, a redundant role of these systems has been suggested [28]. Several studies have proposed that NOD receptors cooperate with TLRs, since addition of NOD receptor agonists augments the inflammatory response (specifically, IL-6, IL-8, IL-10, and TNF-α production) of human or murine macrophages or monocytes exposed to TLR ligands [29]–[31]. In contrast, a role for NOD receptors as negative regulators of TLR responses has also been demonstrated, as stimulation of NOD2 by MDP leads to downregulation of TLR2-mediated secretion of the Th1-promoting cytokine IL-12 [32]. These different cellular responses to TLR and NOD receptor costimulation suggest that, depending on the ligand and inflammatory response studied, activation of NOD receptors can have either a positive or negative regulatory effect on TLR responses.

**Figure 2:**
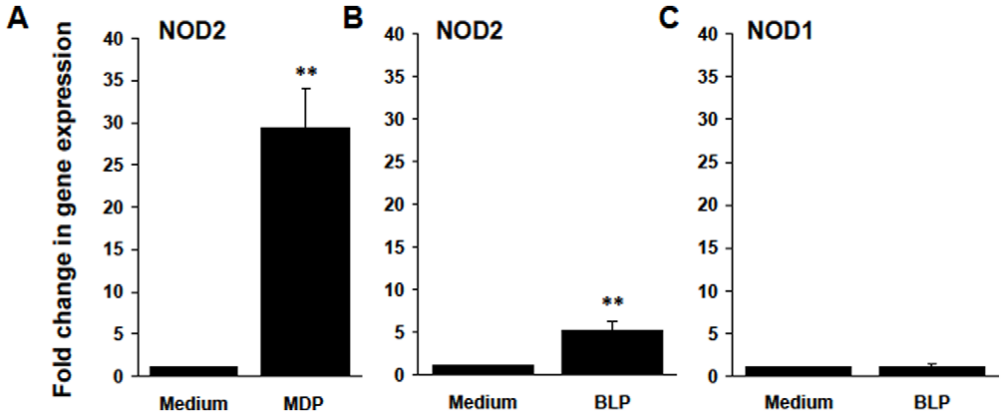
NOD1 and NOD2 mRNA levels in RACs. RACs cultured for 3 passages were treated with medium or 1 μg/ml of MDP (A) or 0.1 μg/ml of BLP (B and C) for 24 h, then the cells were collected and NOD1 (C) and NOD2 (A and B) mRNA levels were determined by RT-qPCR. **p<0.01 compared with RAC treated with medium in Student's *t* test.

The regulation of NODs and the interaction of NODs with TLR2 on RACs have not been studied. As potential APCs in the eye, RACs may play a critical role in host defense by priming immune responses and also contribute to adaptive immunity. In the present study, we examined the influence of NOD2 on the activation of RACs and how the interaction of NOD and TLR on RACs affects the disease-inducing ability of uveitogenic T cells.

## Materials and Methods

### Animals and reagents

Pathogen-free female C57BL/6J (B6, stock number 000664) mice and NOD2-deficient mice on the B6 background (stock number 005763) purchased from Jackson Laboratory (Bar Harbor, ME) were housed and maintained in the animal facilities of the University of Louisville. All animal studies conformed to the Association for Research in Vision and Ophthalmology statement on the use of animals in Ophthalmic and Vision Research. Institutional approval was obtained and institutional guidelines regarding animal experimentation followed. The mouse TLR1/2 agonist BLP (Pam3CSK4) and the NOD2 ligand MDP were obtained from Invivogen (San Diego, CA). The RIP2 inhibitor SB203580 and the IRAK1/4 inhibitor were purchased from Sigma (St. Louis, MO) and EMD Chemicals (Gibbstown, NJ), respectively.

**Figure 3:**
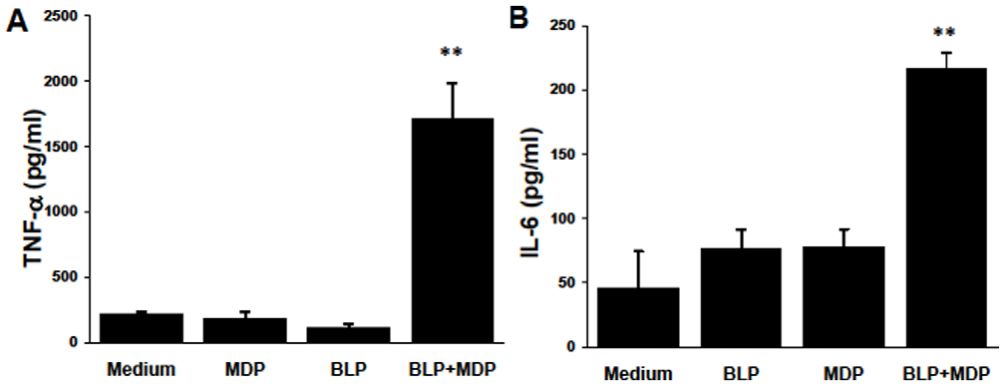
BLP and MDP synergistically enhance cytokine production by RACs. RACs were incubated with medium, MDP (1 μg/ml), BLP (0.1 μg/ml), or MDP plus BLP for 12 h, then the supernatants were collected for measurement of TNF-α (A) and IL-6 (B) by ELISA. The results are the mean ± SEM for three independent experiments, each in duplicate. **p<0.01 compared with RAC treated with medium, BLP or MDP alone in one-way ANOVA.

### Isolation and culture of primary RACs and RPE cells

The methods for the isolation of RACs and RPE cells have been described previously [14]. In detail for RAC isolation and characterization, single retinal neuronal cells were incubated for two weeks on poly-D-lysine–coated six-well plates, with the plates shaken for 2 hrs at room temperature. The supernatant, containing floating dead cells and possible microglia, was discarded and a low concentration (0.05%) of EDTA trypsin added to adherent cells additional shaking for 40 min. The cells removed by the low concentration of EDTA trypsin were collected and transferred to a new flask. These cells were stained with Abs specific for glial fibrillary acidic protein (GFAP, Sigma-Aldrich), S-100 (Santa Cruz Biotechnology, Santa Cruz, CA), vimentin (Santa Cruz Biotechnology, Santa Cruz, CA), retinaldehyde-binding protein (CRALBP, Abcam, Cambridge, MA) and glutamine synthetase (GS, Abcam) followed by analysis under fluorescence microscopy. The phenotype of transferred cells which we used in this study was >95% positive for astrocytes markers (GFAP and S-100), but negative for presumable markers (CRALBP, vimentin, GS) for Müller cells [33], [34] (Fig. 1).

**Figure 4:**
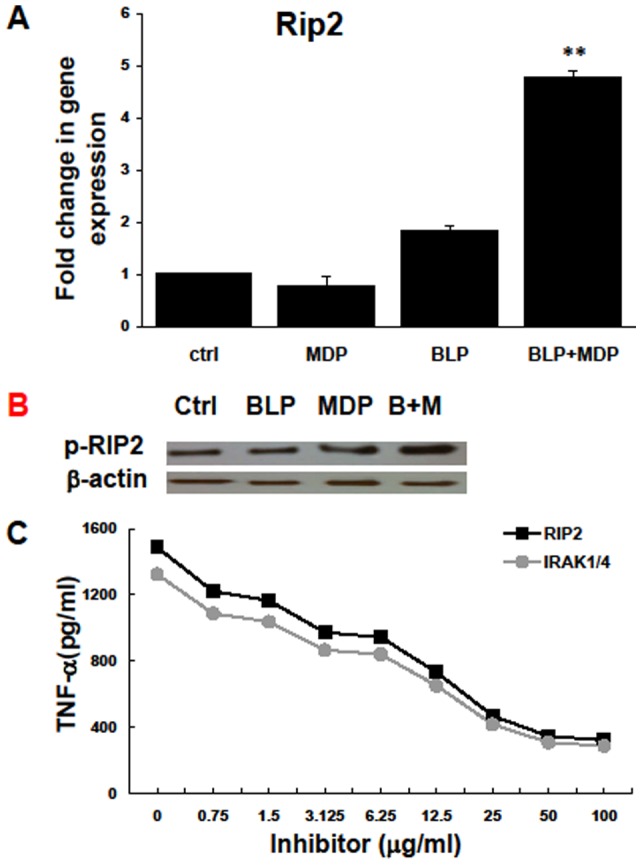
MDP plus BLP increases Rip2 kinase expression. (A) RACs were treated as described in Fig. 2 for 24 h, then the cells were collected and Rip2 mRNA levels determined by RT-qPCR. **, p<0.01, compared with RAC treated with medium, BLP or MDP alone in one-way ANOVA (B) RACs were treated for 15 minutes with medium, BLP, MDP or BLP+MDP, and then the cells were harvested for protein extraction and Western blot analysis using anti-phospho-Rip2 (Ser176) or anti-β-actin antibodies. (C) RACs were pre-incubated with medium alone or medium containing an inhibitor of Rip2 or IRAK1/4 at the indicated concentration for 2 h, then were incubated with BLP plus MDP for 24 h and the supernatant collected for TNF-α measurement by ELISA. The data presented are the mean value (+SD) for triplicate samples for each concentration.

The purity of RPE cells was >95%, as assessed by staining with anti-pan keratin antibody (clone PCK-26, Sigma-Aldrich) and anti-RPE65 antibody (Novus, Littleton, CO, USA) [14]. RACs and RPE cells were used in experiments at three to five passages.

### Actively induced and adoptively transferred experimental uveitis in B6 mice

For active induction of disease, the animals were immunized subcutaneously with 100 μl of an emulsion containing human IRBP1-20 (150 μg) and 500 μg of Mycobacterium tuberculosis H37Ra (Difco, Detroit, MI) in incomplete Freund's adjuvant (Sigma, St Louis), distributed over six spots on the tail base and flank. Concurrently, 0.2 μg of pertussis toxin was injected intraperitoneally (i.p.).

**Figure 5:**
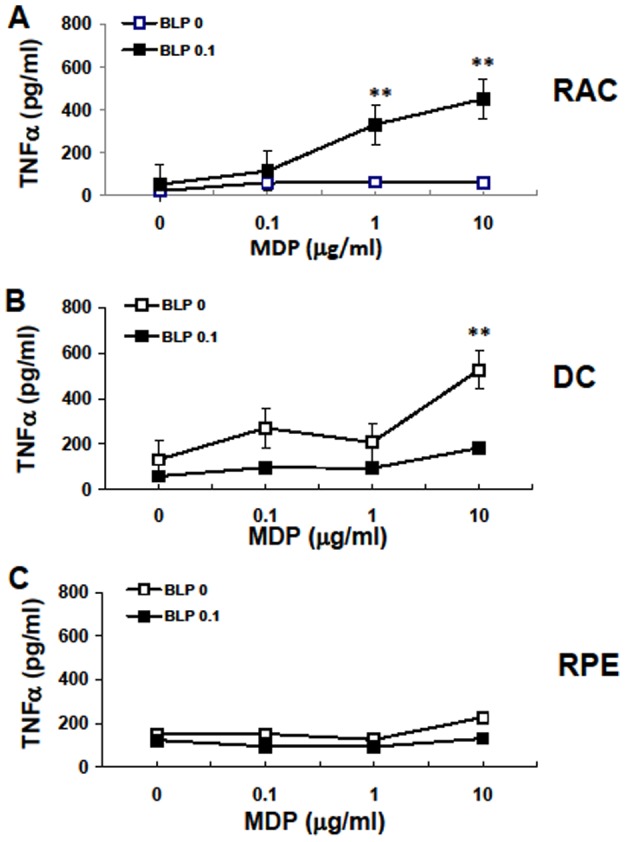
The synergistic effect of BLP and MDP on TNF-α production is cell-type specific. RACs, RPE cells, or bone marrow derived DC were incubated with increasing concentrations of MDP in the presence or absence of BLP (0.1 μg/ml) for 12 h, then TNF-α in the culture supernatants was measured by ELISA. The results are the mean ± SEM for three independent experiments, each in duplicate. *p<0.05, **p<0.01 compared with cells treated with medium or MDP alone at different doses in two-way ANOVA with Fisher LSD test.

For adoptive transfer, recipient animals were injected i.p. with 0.2 ml of phosphate-buffered saline containing 5×10^6^ IRBP1-20-specific T cells, prepared as described previously [35], [36], but using RACs as APCs.

The clinical course of the disease was assessed by indirect fundoscopy twice a week and graded as described previously [37]. The pathology was confirmed by histology [36].

### Preparation of IRBP1-20-specific T cells

**Figure 6:**
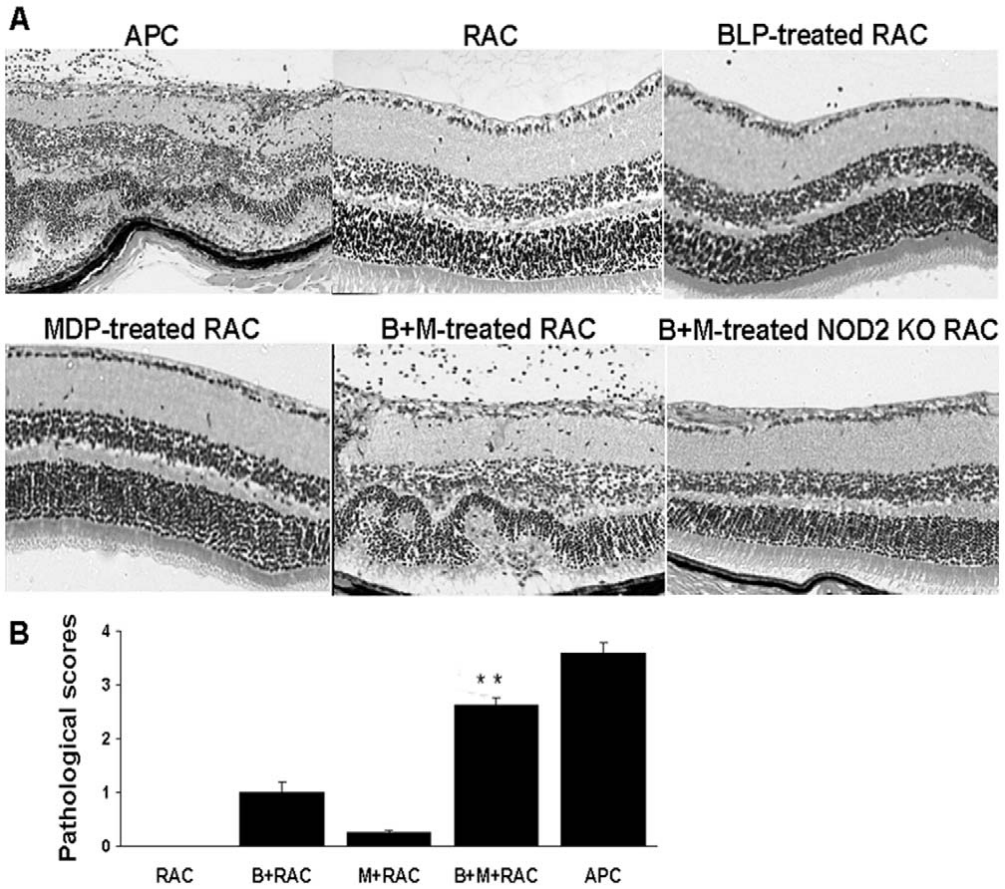
BLP and MDP pre-treated RACs enhance the disease-inducing ability of IRBP-specific T cells. IRBP1–20-specific T cells from the draining lymph nodes and spleens of donor B6 mice immunized with IRBP1–20 were stimulated with IRBP1–20 and irradiated naïve splenic APCs or RACs from either wild-type B6 or NOD2 knockout mice that had been left untreated or had been pre-treated with BLP and/or MDP as described in Fig. 2, then the T cell blasts (5×10^6^ cells/mouse) were transferred to naïve mice (n = 6/group). Eyes from the recipient mice were collected for histological evaluation on day 18 post-transfer. Histopathological lesions in the eyes of one representative mouse in each group (A) and average disease scores of both eyes in 6 individual mice that received T cells (*B*) are shown. **p<0.01 compared with RAC treated with medium, BLP, or MDP alone using Mann-Whitney U test.

IRBP1-20-specific T cells were prepared from IRBP1-20-immunized mice as described previously [38]. Briefly, T cells were isolated from the lymph node or spleen of B6 mice at 13 days postimmunization by passage through a nylon wool column. The cells (1×10^7^) were stimulated with 20 µg/ml of IRBP1-20 in 2 ml of complete medium (RPMI 1640 medium containing 10% fetal bovine serum, 2 mM glutamax II, 100 IU/mL of penicillin, and 100 µg/mL of streptomycin; Sigma) in a six-well plate (Costar, Cambridge, MA) in the presence of 2×10^7^ irradiated syngeneic spleen cells as APCs. After 2 days, the activated lymphoblasts were isolated by gradient centrifugation in Lymphoprep (Robbins Scientific, Mountain View, CA) and cultured in RPMI 1640 medium (Mediatech Inc, Manassas, VA) supplemented with 20 U/ml of IL-2 (BioLegend, San Diego, CA).

### Generation of bone marrow-derived dendritic cells

**Figure 7:**
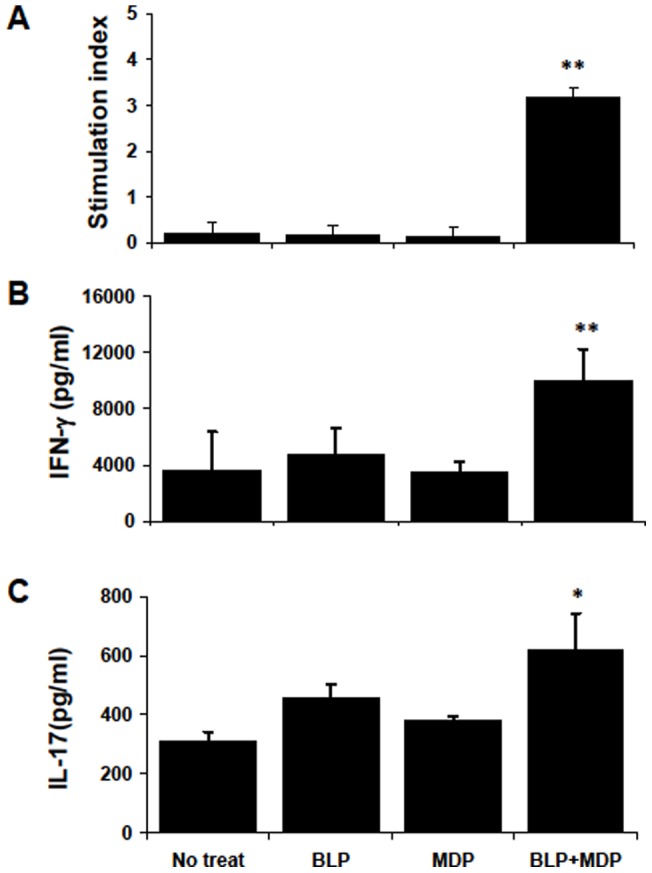
RACs activated by MDP plus BLP induce antigen-specific uveitogenic T cell proliferation and differentiation into Th1and Th17 cells. (A): MDP plus BLP-treated RACs are effective in presenting uveitogenic peptide, leading to the proliferation of IRBP1–20-specific T cells. T cells from in vivo IRBP1–20-primed B6 mice at day 12 were cultured with RACs in the presence of IRBP1–20 and proliferation was measured. Prior to co-culture with T cells, cultured RACs were treated for 24 h with BLP and/or MDP, washed, and treated with MMC. (B & C): MDP plus BLP-pre-treated RACs induce IRBP-specific T cells to produce pro-inflammatory cytokines. The experimental paradigm was as in (A), but, after 48 h, cytokines in the supernatants were measured by ELISA. The values are the mean ± SEM for three individual experiments. *p<0.05, **p<0.01 compared with RAC treated with medium, BLP or MDP alone in one-way ANOVA.

Femurs and tibiae of 6- to 8-week-old B6 mice were aseptically removed and cleared of surrounding muscle, then the bones were cut up and placed in cold complete medium and the bone marrow flushed out with a syringe. Bone marrow cells (1×10^6^ cells per well) in 1 ml of complete medium containing 10 ng/ml of GM-CSF (R&D Systems, Inc., Minneapolis, MN) were plated in 24-well plates and fed every other day. On day 6, non-adherent cells were collected for phenotyping. The purity of the bone marrow DCs determined by staining with anti-CD11c antibody (Biolegend, San Diego, CA ) and flow cytometry analysis was >95%.

**Figure 8:**
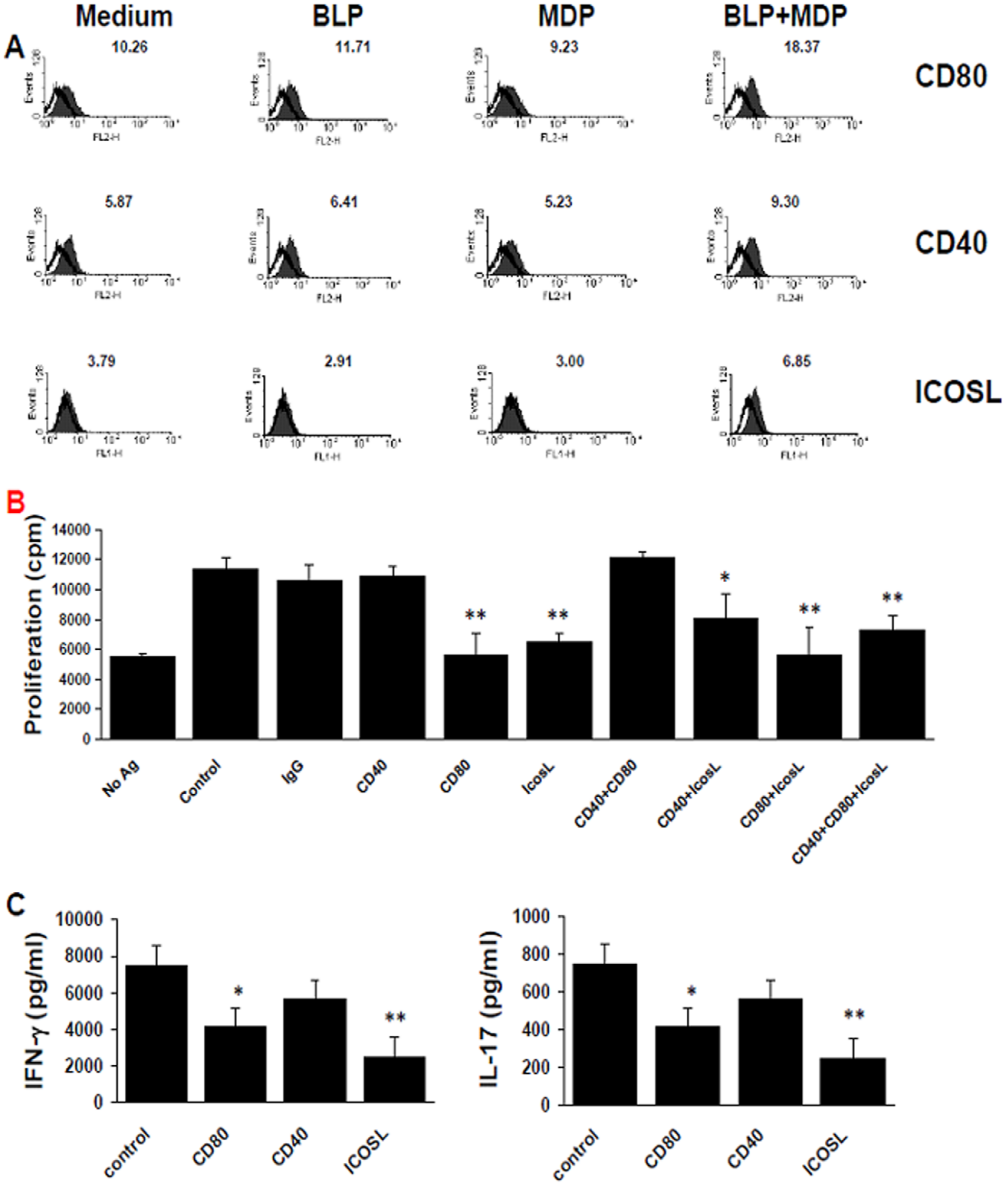
BLP plus MDP induces RACs to express costimulatory molecules. (A) RACs were incubated for 48 h in, from left to right, medium alone or medium containing BLP or MDP or both, then were stained with monoclonal Abs against CD80, CD40, or ICOSL, and analyzed by flow cytometry. Negative control samples were stained with an isotype-matched control IgG Ab (empty filled). Numbers in each histogram represent mean fluorescence intensity values. One of three reproducible experiments is shown. (B) The antigen-specific uveitogenic T cell expansion induced by MDP plus BLP-pre-treated RACs is inhibited by anti-ICOSL and anti-CD80 Abs. T cells from in vivo IRBP1-20-primed B6 mice at day 12 were cultured with MDP plus BLP-pre-treated RACs and IRBP1-20 in the absence or presence of 10 μg/ml of anti-CD80, CD40, and ICOSL Abs either alone or in combination or their rat IgG2a, κ isotype control, then proliferation by the responder T cells were determined. C: Cytokine production of the antigen-specific uveitogenic T cells induced by MDP plus BLP-pre-treated RACs is inhibited by anti-ICOSL and CD80 Abs. The experimental paradigm was as in (B), but, after 48 h, cytokines in culture supernatants were measured by ELISA. The values are the mean ± SEM for three individual experiments. *p<0.05, **p<0.01 compared with control in one-way ANOVA.

### Real-time quantitative RT-PCR assay of NOD1 and NOD2 expression

Total RNA from RACs was extracted using an RNA isolation kit (Invitrogen, Carlsbad, CA), treated with DNase I (GE Healthcare, Piscataway, NJ), and reverse transcribed into cDNA using an MMLV-RT kit (Invitrogen). Each cDNA sample was amplified for the gene of interest and ß-actin (*Taq*Man assays; Mx3000P system; Stratagene, La Jolla, CA). The concentration of the gene of interest was determined using the comparative threshold cycle number and normalized to that of the internal ß-actin control. The primers and probes used were: ß-actin, forward primer, 5′-ATCTACGAGGGCTATGCTCTCC-3′, reverse primer, 5′-ACGCTCGGTCAGGATCTTCAT-3′; NOD1, forward primer, 5′-ACTCAGCG-TCAACCAGATCAC-3′, reverse primer, 5′-ACGATGGAGGTGCTGTTCTTC-3′; NOD2, forward primer, 5′-CTCAGTCTCGCTTCCTCAGTAC-3′, reverse primer, 5′-TGCAGA-AGAGTGCTCTTGCC-3′; and Rip2, forward primer, 5′-TCCAGAGTAAGAGGGAAGC-

C-3′, reverse primer, 5′-TTGGATGTCAGACGTATCTAGC-3′.

### Co-culture of RACs and T cells and measurement of cytokines released by the T cells

The RAC monolayer was incubated for 48 h with IRBP1-20-specific T cells (3×10^5^) in the presence of IRBP1–20, then the culture supernatants were collected for cytokine assay by ELISA (R&D Systems, Minneapolis, MN). To exclude the possibility that the cytokines were produced by the astrocytes, rather than the T cells, the astrocytes were treated with 100 μg/ml of mitomycin C (MMC; Sigma-Aldrich Co. LLC. St. Louis, MO) for 1 h at 37°C before being mixed with the responder T cells [14], [39].

### Proliferation assay

T cells from IRBP1–20-immunized B6 mice were prepared and seeded at 4×10^5^ cells/well in 96-well plates, then cultured at 37°C for 72 h with or without IRBP1–20 in the presence of irradiated syngeneic spleen APCs (1×10^5^) or MMC-treated RACs in a total volume of 200 µl of complete medium and [^3^H]thymidine incorporation during the last 8 h was assessed using a microplate scintillation counter (Packard Instrument). The proliferative response was expressed as the mean cpm ± SD of triplicate determinations. In blocking experiments, functional grade purified Abs (eBioscience) against mouse CD40 (clone number: 1C10), CD80 (clone number 16-10A1), ICOSL (clone number: HK5.3) and their common isotype control rat IgG2a, κ were used at dose of 10 μg/ml.

### Flow cytometry analysis

RACs or T cells were incubated for 30 min at 4°C in staining buffer (PBS containing 3% fetal calf serum and 0.1% sodium azide) containing phycoerythrin- or fluorescein isothiocyanate-conjugated Abs (eBioscience) against mouse CD80, ICOSL, CD40 or their rat IgG2a, κ isotype control. The cells were then washed, re-suspended in staining buffer, and analyzed by flow cytometry (FACS_Calibur_, BD) using Cellquest software.

### Western blot analysis

Confluent RACs in a 75 cm plate were stimulated with 0.1 µg/ml of BLP and/or 1 μg/ml of MDP in fresh 3% complete medium for 15 min, then cell lysates were prepared using RIPA buffer [(50 mM Tris pH 8.0, 150 mM NaCl, 1.0% IGEPAL® CA-630, 0.5% sodium deoxycholate, 0.1%SDS (Sigma)] and run on SDS polyacrylamide gels. Western blotting was performed on nitrocellulose membranes (Bio-Rad) as described previously [40] using antibody against phosphorylated RIP2 (R&D Systems, Minneapolis, MN).

### Statistical analysis

Experiments were repeated at least twice, usually three or more times. An unpaired Student's *t* test for two sets of data, one-way or two-way ANOVA for more than two sets of data and Mann-Whitney *U* test for clinical score of uveitis were used for statistical analysis (ProStat Ver 5.5 software). Values determined to be significantly different from those for controls are marked with an asterisk in the figures (*: *p*<0.05, **: *p*<0.01).

## Results

### Expression of NOD2 and NOD1 mRNAs by cultured B6 RACs

We have previously reported that mouse RACs express TLR2, TLR3, and TLR4 [15]. To determine whether RACs expressed NODs, we isolated RACs from the retina of naïve B6 mice as described previously [14] and measured levels of NOD1 and NOD2 mRNAs using RT-PCR. As shown in Fig. 2A–C, resting RACs expressed low levels of both mRNAs and NOD2 mRNA levels were dramatically increased by stimulation with the NOD2 ligand MDP (Fig. 2A) or the TLR2 ligand BLP (Fig. 2B). BLP did not upregulate NOD1 mRNA expression (Fig. 2C).

### Synergistic effects of BLP and NOD2 on RAC production of cytokines

We have previously reported that BLP has a weaker activating effect on RACs than Poly I:C and LPS. To examine the effect of NOD2 and its possible interplay with TLR2 on RACs, we exposed RACs to BLP and/or MDP for 24 h and measured TNF-α or IL-6 production. As shown in Fig. 3, the results agreed with our previous observation that exposure to BLP alone did not induce TNF-α production by RACs [15] and also showed that MDP alone did not induce RACs to produce TNF-α. However, co-incubation of RACs with MDP plus BLP significantly enhanced TNF-α and IL-6 production compared with BLP or MDP alone (Fig. 3).

### Rip2 activation in RACs stimulated with MDP plus BLP

Rip2 is the downstream signaling pathway molecule for NOD2. To determine whether the synergistic effect of MDP plus BLP on RAC activation was associated with the NOD2 signaling pathway, Rip2 mRNA levels were quantitatively assessed in RACs pre-treated with MDP and/or BLP. As shown in Fig. 4A, Rip2 mRNA levels were greatly increased by pre-treatment with MDP plus BLP compared to treatment with MDP or BLP alone. Moreover, phosphorylation of Rip2 was increased in RACs pre-exposed to MDP plus BLP (Fig. 4B). As shown in Fig. 4C, the Rip2 inhibitor SB203580 significantly inhibited TNF-α production by RACs pretreated with MDP plus BLP in a dose-dependent manner, and similar inhibition was seen using an antagonist of IRAK1/4, protein kinases that mediate signaling by IL-1, IL-18, and TLRs.

### The response of RACs to NOD2 ligand differs from those of bone marrow DCs and RPE cells

To determine whether the response of RACs to MDP and/or BLP was cell type-specific, we incubated RACs, RPE cells, and bone-marrow-derived DCs for 24 h with MDP (0.1–10µg/ml) and/or BLP (0.1 µg/ml), then measured TNF-α in the culture supernatants by ELISA. As shown in Fig. 5A, BLP or MDP alone did not induce RACs to produce TNF-α, whereas a marked MDP dose-dependent increase in TNF-α production was seen using BLP and MDP. In contrast, bone-marrow-derived DCs responded to MDP alone in a dose-dependent manner and addition of BLP inhibited the response to MDP (Fig. 5B), while RPE cells responded poorly to BLP or NOD2 alone or in combination (Fig. 5C).

### MDP and BLP synergistically enhance the stimulatory effect of RACs on the ability of IRBP1-20-specific T cells to induce EAU

To determine whether the synergistic effect of MDP and BLP altered the stimulatory action of RACs on IRBP-specific T cells, we pre-treated RACs with MDP and/or BLP then co-cultured them with IRBP-specific T cells for 48 h and tested the T cells for uveitogenic activity by adoptive transfer to naïve mice. As shown in Fig. 6A and B, T cells stimulated by MDP-treated or BLP-treated RACs either failed to induce uveitis or induced mild uveitis in recipient mice, respectively, whereas those stimulated by BLP plus MDP-treated RACs were effective in disease induction. Histopathological analysis of eyes collected 18 d after adoptive transfer showed cellular infiltrate in the posterior segments of the eyes and a partial or complete destruction of the photoreceptor cells. In addition, the synergistic stimulatory effect of MDP and BLP on RACs was lost when the RACs were derived from NOD2-deficient mice (Fig. 6A).

To determine how MDP plus BLP-treated RACs increased the disease-inducing ability of IRBP-specific T cells, IRBP1–20–specific T cells were assessed for proliferation and cytokine production after incubation with RACs pre-treated with BLP and/or MDP. As controls, the same responder T cells were incubated with IRBP1–20 in the presence of B6 splenic APCs. As shown in Fig 7, untreated RACs or RACs pre-treated with MDP or BLP alone had no stimulatory effect on T cells in terms of proliferation (Fig. 7A) or production of IFN-γ (Fig. 7B) or IL-17 (Fig. 7C), whereas stimulation of all three activities was seen in IRBP1–20-specific T cells cultured with MDP plus BLP-treated RACs.

### MDP and BLP increase the expression of costimulatory molecules on RACs

T cell activation requires costimulatory molecules. We therefore measured the expression of co-stimulatory molecules by RACs before and after exposure to MDP and/or BLP. As shown in Fig. 8A, the combination of MDP plus BLP induced higher levels of CD80, CD40, and ICOSL on RACs, whereas BLP or MDP alone did not. In addition, as shown in Fig. 8B and C, blockade of CD80 or ICOSL by Abs prevented the stimulatory effect of MDP plus BLP-treated RACs on proliferation of IRBP-specific T cells and production of IFN-γ and IL-17, whereas blockade of CD40 did not. The non-inhibitory effect of anti-CD40 Ab on responder T cell proliferation was dominant when in combination with anti-CD80 Ab, but not with anti-ICOSL Ab (Fig. 8B).

## Discussion

Pathogen-recognizing receptors, including TLRs and NODs, are primarily expressed by myelomonocytic cells and DCs. We have previously reported that mouse retinal astrocytes, potential APCs in the eye, express TLRs, such as TLR2, TLR3, and TLR4; and that stimulation of RACs with TLR3 or TLR4 ligands upregulates production of IL-6, TNF-α, IL-23, and IL-12 [15], suggesting that RACs are able to respond to microbial proteins or endogenous TLR ligands and actively participate in inflammatory responses during pathogen-host interactions. In the present study, we also found that RACs express a second family of PRRs, NOD1 and NOD2. Resting RACs constitutively expressed low levels of NOD2 mRNA and, after incubation with the NOD2 ligand MDP, NOD2 mRNA expression was significantly increased (Fig. 2A). The expression of NODs on RACs indicates that RACs might be able to recognize distinct substructures of PGN present in Gram-negative and Gram-positive bacteria and produce antimicrobial factors. PGN is found not only in the normal mucosal flora from the gut and infection, but also in the gut epithelium and within DCs in secondary lymphoid organs [41], [42], human spleen [43], and in the central nervous system of primates with multiple sclerosis and experimental allergic encephalomyelitis [27], [44].

We also showed that TLR ligands such as bacterial ligands for TLR2 (Fig. 2B) and TLR4 (data not shown) or virus dsRNA (Poly I:C) a TLR3 ligand, (data not shown) upregulated NOD2 mRNA expression in RACs. This phenomenon has also been documented in other cell types. For example, LPS, (a TLR4 ligand), purified flagellin (Flg, a TLR5 ligand), or an activating oligonucleotide CpG (a TLR9 ligand) increase NOD2 mRNA levels in isolated murine brain astrocytes [45] and LPS induces NOD2 mRNA expression in a monocytic cell line [46]. Resting astrocytes expressed low levels of NOD1 mRNA, but, unlike that of NOD2, NOD1 expression was not increased by TLR ligands (Fig. 2C). In brain astrocytes, increased NOD1 expression is observed when they are exposed to LPS, Flg, or CPG, but this increase is modest compared to the increase in NOD2 expression after challenge with these TLR ligands [45]. The upregulation of NOD2 gene expression by bacterial components or inflammatory cytokines might be explained by the facts that the promoter region of NOD2 contains a NF-κB-consensus sequence [28], [47] and that, although triggering events of the signaling pathways for TLRs and cytokines TNFα/IFN-γ are different, they both result in NF-κB activation [28], [47]. Because NOD2 activates NF-κB and this response is likely to mediate the induction of cytokines, including TNFα, upregulation of NOD2 may be part of a positive regulatory loop involving inflammatory cytokines or bacterial components.

MDP or BLP individually had little effect on RAC activation, but, in combination, they had a strong stimulatory effect (Fig. 3). These results indicate that RACs have the ability to recognize PGN by both TLR2 and NOD2. At the molecular level, the induction of the synergistic effects seemed to be mediated, at least in part, by increased levels of phosphorylated Rip2 (Fig. 4B), a serine/threonine kinasehttp://www.nature.com/nature/journal/v416/n6877/full/416194a.html – B1#B1 essential for the activation of NF-κB by NOD1 and NOD2 and also required for optimal TLR signaling [20]. Since either Rip2 or IRAK inhibitors could inhibit TNF-α production by BLP plus MDP-treated RACs in a dose dependent manner (Fig. 4C), it suggests that both TLR (MyD88-IRAK) and NOD (Rip2) signaling pathways are mutually involved in RAC activation. However, the mechanisms by which they cross-talk among different PRRs remain to be further investigated.

This synergistic effect seemed to be RAC-specific, as, in bone marrow-derived DCs, MDP alone induced TNF-α production in a dose-dependent manner and addition of BLP had an inhibitory effect (Fig. 5B), while RPE cells did not response to stimulation by BLP or MDP alone or in combination (Fig. 5C). The low responsiveness of RPE cells to inflammatory stimulation helps in their role of keeping the eye in an immune privileged state [48], [49] and ensuring the proper function of the eye. The responses of different cell types to TLR2 and NOD2 activation are varied. For example, incubation of splenocytes with BLP plus MDP increases TNF secretion, but reduces IL-12 secretion, as TLR2-induced IL-12 production is inhibited by MBP [32]. The different interplay between TLR2 and NOD2 in different immune cell types might be explained by their functions in the immune system, as DCs are professional APCs involved in peripheral naïve T cell activation, whereas RACs are parenchymal cells that protect the eye from inflammation and injury. The characterization of the responses of different cell types to TLR and NOD ligands should increase our understanding of the contribution of each cell to the innate host response to infection or tissue damage.

The synergistic effect of TLR2 and NOD2 ligands on RACs was also seen as increased expression of positive costimulatory molecules that are required for T cell activation (Fig. 8). CD80 or ICOSL seems functionally important for RAC to stimulate responder T cells. The observations that incubation of IRBP-specific T cells with RACs pre-exposed to BLP plus NOD2 led to the production of higher levels of IFN-γ and IL-17 by IRBP1-20-specific T cells and that adoptive transfer of these cells into naïve mice induced severe uveitis suggests that the stimulation of RACs via both the NOD2 and TLR2 pathways is a more potent inducer of the APC function of RACs than either ligand alone and that a variety of infectious pathogens may have the ability to stimulate RACs and contribute to the development of autoimmune uveitis.

In summary, our studies demonstrate that the stimulatory effect of RACs on autoreactive T cells can be regulated by ligands of pathogen recognition receptors including TLRs and NODs. Previous publication [15] showed LPS or PolyI:C alone had a marked effect on the ability of RACs to promote the activation of Th1 and Th17 IRBP-specific T cells, whereas current work showed that two weak stimulators of TLR1/2 and NOD2 together could augment their effects on RAC activation, thus, these RACs acquired the ability to enhance pathogenecity of uveitogenic T cells.
